# Oxytocin and Gynecomastia: Correlation or Causality?

**DOI:** 10.7759/cureus.2661

**Published:** 2018-05-21

**Authors:** Leticia Amorim, Flavia V Gouveia, Jürgen Germann, Debora Zambori, Rosa Morais, Flavia M Sato, Camila Fongaro, Joana Portolese, Helena Brentani, Raquel Martinez

**Affiliations:** 1 Instituto De Psiquiatria, Hospital das Clinicas da Faculdade de Medicina da Universidade de São Paulo, São Paulo, BRA; 2 Instituto De Ensino E Pesquisa, Hospital Sirio Libanes; 3 Douglas Mental Health University Institute, McGill University Health Centre, Montreal, QC, Canada., Montreal, CAN

**Keywords:** oxytocin, chronic administration, autism, gynecomastia, side effects

## Abstract

Oxytocin has been administered to patients with autism spectrum disorder (ASD) in order to improve social skills, communication, and manage repetitive behaviors in the context of research trials. The majority of the studies focus on acute administration; thus, the effectiveness and potential side effects of chronic administration remain unknown. The main goal of this case report is to highlight the importance of the safety parameters for the chronic use of intranasal oxytocin administration. In a single case conducted in our outpatient clinic, one adolescent (15 years old) received intranasal oxytocin (24 IU) twice per day, in accordance with the recommended doses for this age group that varies from 8 - 25 IU twice per day. After three weeks of treatment, the patient presented with gynecomastia. While it is not certain that the gynecomastia was oxytocin-induced, this case highlights the importance of developing optimal regimens for chronic oxytocin administration, with a particular focus on safety parameters.

## Introduction

Autism spectrum disorder (ASD) is a neurodevelopmental disorder that includes a group of complex and prevalent impairments in social interaction and verbal and nonverbal communication; patients with ASD often have restricted interests and stereotyped behaviors [1]. A systematic review of previous randomized trials in patients with ASD treated with intranasal oxytocin administration have found promising results with regard to eye contact and emotion recognition measures; oxytocin was well tolerated and side effects were described as mild [2].

Concerning the safety aspects of long-term oxytocin administration, the side effects reported in the literature included mild nose irritation/congestion, increased day/nighttime urination, diarrhea or constipation, fatigue, irritability, worsening tics, leg shaking, increased energy, loss of appetite/weight, increased thirst, trouble sleeping, increased acne, and a slightly elevated temperature [3-6]. The serious adverse reactions observed included hyperactivity and aggression (n = 3/31), which excluded the participants from continuing the study; those symptoms ceased once the oxytocin treatment was discontinued [6]. Munesue et al. reported that one patient had an epileptic seizure that was attributed to forgetting to take antiepileptic medication [7]. Another study reported no noteworthy side effects after chronic administration [8]. In a systematic safety review of 1529 participants, 279 cases of mild adverse reaction to oxytocin were reported, such as drowsiness/sleepiness, dry throat/mouth, nasal irritation, runny nose, abdominal/stomach pain, anxious/worried/uncomfortable, and headache [9]. The described severe side-effects due to long-term use included water intoxication due to excessive water intake, psychotic symptoms, lightheadedness/vertigo, and memory loss, specifically, severe temporary anterograde memory disturbance.

A better understanding of the side effects of long-term administration is necessary to assess the safety of chronic intranasal oxytocin administration in patients with ASD.

## Case presentation

**Figure 1:**
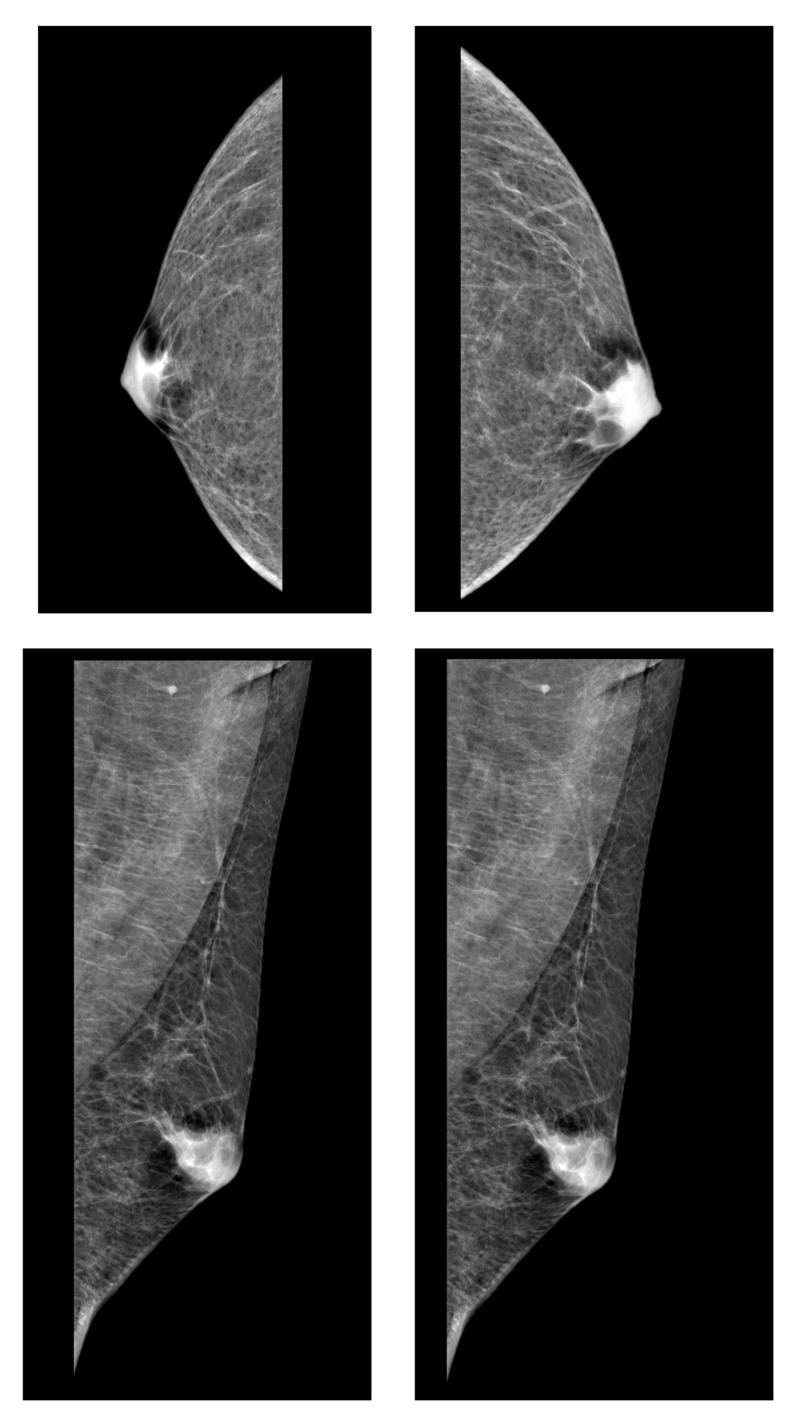
Digital mammography Digital mammography revealed an enlargement of the breast resulting from a proliferation of the glandular component, with the presence of a firm mass concentrically from the nipples, confirming the gynecomastia.

In March 2016, an ASD male patient from our outpatient clinic began to use intranasal oxytocin (24 IU) twice per day. The patient baseline characteristics were 15 years old, Childhood Autism Rating Scale (CARS) = 32, Autism Diagnostic Interview (ADI) = 52, intelligence quotient (IQ global - Wechsler Intelligence Scale for Children (WISC)) score = 112, and no use of psychotropic medication. The participant’s legally authorized representative was informed and asked to sign the free and informed consent. The project was approved by the Ethics in Research Committee of the Faculty of Medicine, USP / Brazil Platform: CAAE 10922213.7.0000.0068 and registered in ClinicalTrials.gov, Identifier: NCT02007447. Routine physical examination after three weeks of oxytocin administration revealed an enlargement of the breasts resulting from a proliferation of the glandular component, with the presence of a firm mass concentrically from the nipples, suggesting bilateral gynecomastia; digital mammography confirmed the diagnosis (Figure 1).

Furthermore, after the onset of the adverse event, the oxytocin treatment was discontinued but the current status is that the gynecomastia has persisted. The puberty of the ASD patient started with 12 years old, and there was no history of endocrinological disorders.

Table 1 summarizes the principal results from the case report. For the Aberrant Behavior Checklist (ABC), the value dropped from 49 (Week 1) to 19 (Week 3; mean ± standard deviation (SD): 35.7 ± 15.3). The Clinical Global Impression - Severity (CGI-S) and Clinical Global Impression - Improvement (CGI-I) values showed minor changes over the course of treatment (mean ± SD: 3.3 ± 0.6). The Repetitive Behavior Scale (RBS) decreased from 22 (Week 1) to 10 (Week 3) (mean ± SD: 14.7 ± 6.4). The data only correspond to Weeks 1-4 because the patient presented with adverse effects and had to stop the study. Unfortunately, the Matson Evaluation of Social Skills with Youngsters (MESSY), Vineland Adaptive Behavior Scale, and Multidimensional Student’s Life Satisfaction Scale (MSLSS) tests were evaluated just once. The intranasal administration of oxytocin had no significant effect on the bloodwork, including electrolytes, liver/renal function, fat metabolism, glucose, triglycerides, blood cell types, thyroid function, testosterone levels, prolactin, serum cortisol, weight (body weight and body mass index), and cardiovascular parameters (body pressure and pulse rate in two different positions: lying down and standing up). 

**Table 1:** Summary of the Principal Results from the Case Report Data from several parameters measured one, two, and three weeks after the administration of oxytocin. ABC: Aberrant Behavior Checklist; CGI-S: Clinical Global Impression-Severity; CGI-I: Clinical Global Impression-Intensity; MESSY: Matson Evaluation of Social Skills with Youngsters; RBS: Repetitive Behavior Scale; Vineland: Vineland Adaptive Behavior Scale; MSLSS: Multidimensional Student’s Life Satisfaction Scale. HDL: high-density lipoprotein; LDL: low-density lipoprotein; VLDL: very-low-density lipoprotein; TSH: thyroid-stimulating hormone; T3: triiodothyronine; T4: free thyroxine; SHBG: sex hormone-binding globulin. BMI: body mass index; BP lying down: Blood pressure reading when the person was lying down; BP standing up: Blood pressure reading when the person was standing up; Pulse lying down: Pulse rates evaluated when lying down; Pulse standing up: Pulse rates evaluated when standing up; Temperature: body temperature; *: Patient was excluded for presenting adverse effect

Parameters	Week 1	Week 2	Week 3
ABC	49	39	19
CGI-S	3	4	3
CGI-I	4	3	3
Messy	143	----	----
RBS	22	12	10
Vineland	9	----	----
MSLSS	172	----	----
Sodium (mEq/L)	138	----	138
Potassium (mEq/L)	4.4	----	4.3
Amylase (U/L)	55	----	62
Phosphatase alkaline (U/L)	137	----	112
Gamma-glutamyl transpeptidase (U/L)	15	----	16
Aspartate aminotransferase (U/L)	16	----	18
Alanine aminotransferase (U/L)	16	----	19
Creatine (mg/dL)	0.64	----	0.65
Urea (ng/dL)	22	----	27
Total cholesterol (mg/dL)	133	----	143
LDL cholesterol (mg/dL)	85	----	85
VLDL cholesterol (mg/dL)	12	----	11
HDL cholesterol (mg/dL)	36	----	36
Glucose (mg/dL)	84	----	92
Triglycerides (mg/dL)	62	----	57
Hemoglobin (g/dL)	15.4	----	15.6
Hematocrit (%)	45.7	----	45.8
Leukocytes (mil/mm^3^)	9.38	----	6.47
Neutrophils (mil/mm^3^)	5.24	----	3.08
Platelets (mil/mm^3^)	274	----	250
TSH (mIU/L)	2.68	----	3.61
T4-free (ng/dL)	1.30	----	1.33
T4 (mg/dL)	6.9	----	8.0
T3 (ng/dL)	142	----	151
Testosterone (ng/dL)	250	----	316
Testosterone-free (ng/dL)	235	----	300
SHBG (nmol/L)	16.8	----	17.2
Prolactin (ng/mL)	6.2	----	5.6
Serum cortisol (mg/dL)	9.5	----	7.5
Height (m)	1.81	1.81	1.81
Weight (Kg)	80.35	80.80	81.25
BMI	24.57	24.70	25.39
BP lying down (mmHg)	100/60	110/60	110/70
BP standing up(mmHg)	108/80	110/80	110/80
Lying down pulse (bpm)	58	94	60
Pulse standing up (bpm)	80	90	80
Temperature	35.3°C	36°C	36°C

The patient in our study showed gynecomastia after the administration of oxytocin, 24 IU twice-per-day over three weeks - a serious and not yet reported possible side effect - highlighting the importance of better exploring of oxytocin’s safety parameters.

## Discussion

To our knowledge, this is the first study to report gynecomastia after three weeks of twice-a-day administration of intranasal oxytocin (24 UI). The main goal of this case report is to highlight the importance of the safety parameters for the use of intranasal oxytocin administration.

First, care was taken for the chosen oxytocin dose. In our study, we proposed intranasal oxytocin (24 IU), administered twice per day, in ASD teenagers (12 - 17 years) for eight weeks. This choice is based on previous work in which ASD children and adolescents (3 - 18 years old) received intranasal oxytocin administration from 12 to 24 IU twice per day [4, 6].

Because the protocol for chronic oxytocin administration had not been established, in our study, additional safety parameters were evaluated, including bloodwork (electrolytes, liver/renal/thyroid function, fat metabolism, glucose, triglycerides, blood cell types, and testosterone levels), weight measurements (body weight, height, and body mass index), cardiovascular parameters (body pressure and pulse rate), and body temperature. This caution was similar to those of previous studies using oxytocin administration [4-6].

It is challenging to distinguish physiological gynecomastia from those with an underlying pathology. Our patient had no history of endocrinological disorders and the puberty started three years prior. Also, there were no impairments in the levels of estrogen, testosterone, and sex hormone-binding globulin (SHBG). However, gynecomastia could be regarded as a part of normal development in adolescent boys [10]. According to a recent review, the main cause of the gynecomastia is an imbalance between the stimulatory effect of estrogen and the inhibitory effect of androgen [10]. Additionally, oxytocin receptors are widely distributed in the body periphery, especially in the mammary tissue [11]. Therefore, it remains undetermined that the gynecomastia in this patient was caused by oxytocin and remains a possible case of oxytocin-induced gynecomastia.

Further studies that focus on evaluating the side effects of oxytocin treatment are necessary to help understand the complexity of the oxytocinergic system.

## Conclusions

While it is not certain that the gynecomastia described in this case report was oxytocin-induced, this result highlights the importance of optimal regimens of oxytocin administration, including dose, duration, and administration, to monitor all possible side effects in order to guarantee the patient’s safety.
